# Age of onset correlates with clinical characteristics and prognostic outcomes in neuromyelitis optica spectrum disorder

**DOI:** 10.3389/fimmu.2022.1056944

**Published:** 2022-12-08

**Authors:** Yacen Hu, Qiying Sun, Fang Yi, Lingyan Yao, Yun Tian, Haiyun Tang, Mengchuan Luo, Nina Xie, Zhiqin Wang, Xinxin Liao, Lin Zhou, Hongwei Xu, Yafang Zhou

**Affiliations:** ^1^ Department of Geriatric Neurology, Xiangya Hospital, Central South University, Changsha, Hunan, China; ^2^ National Clinical Research Center for Geriatric Disorders, Xiangya Hospital, Central South University, Changsha, Hunan, China; ^3^ Department of Radiology, Xiangya Hospital, Central South University, Changsha, Hunan, China; ^4^ Department of Neurology, Xiangya Hospital, Central South University, Changsha, Hunan, China

**Keywords:** neuromyelitis optica spectrum disorder, late-onset NMOSD, age of onset, clinical characteristic, prognosis

## Abstract

**Objective:**

Neuromyelitis optica spectrum disorder (NMOSD) is an inflammatory disease preferentially affects the optic nerve and the spinal cord. The first attack usually occurs in the third or fourth decade, though patients with disease onset in the fifties or later are not uncommon. This study aimed to investigate the clinical characteristics and prognosis in patients with different age of onset and to explore the correlations between age of onset and clinical characteristics and prognostic outcomes.

**Method:**

We retrospectively reviewed the medical records of 298 NMOSD patients diagnosed according to the 2015 updated version of diagnostic criteria. Patients were divided into early-onset NMOSD (EO-NMOSD) (<50 years at disease onset) and late-onset NMOSD (LO-NMOSD) (≥50 years at disease onset) based on the age of disease onset. LO-NMOSD patients were divided into two subgroups: relative-late-onset NMOSD (RLO-NMOSD) (50~70 years at disease onset) and very-late-onset NMOSD (≥70 years at disease onset). Clinical characteristics, laboratory findings, neuroimaging features, and prognostic outcomes were investigated.

**Results:**

Compared to EO-NMOSD patients, patients with LO-NMOSD showed more frequent transverse myelitis (TM) (58.20% vs. 36.00%, *p* = 0.007) while less frequent optic neuritis (ON) (23.10% vs. 34.80%, *p* = 0.031) and brainstem/cerebral attacks (7.50% vs. 18.30%, *p* = 0.006) as the first attack. Patients with LO-NMOSD showed less frequent relapses, higher Expanded Disability Status Scale (EDSS) score at the last follow-up, fewer NMOSD-typical brain lesions, and longer segments of spinal cord lesions. Patients with older onset age showed a higher proportion of increased protein levels in cerebrospinal fluid during the acute phase of attacks. Age at disease onset positively correlated with length of spinal cord lesions at first attack and at last follow-up, negatively correlated with ARR-1 (ARR excluding the first attack, calculated from disease onset to final follow-up), irrespective of AQP4-IgG serostatus. Patients with older age at disease onset progressed to severe motor disability sooner, and age of onset positively correlated with EDSS score at the last follow-up, irrespective of AQP4-IgG serostatus.

**Conclusion:**

Age of disease onset affects clinical characteristics and prognosis outcomes of patients with NMOSD.

## Introduction

Neuromyelitis optica spectrum disorder (NMOSD) is an inflammatory disease of the central nervous system characterized by recurrent attacks of optic neuritis (ON) and longitudinally extensive transverse myelitis (LETM) (1). (2). The serum aquaporin-4 immunoglobulin G antibody (AQP4-IgG) is considered pathogenic and plays an important role in defining new diagnostic criteria for NMOSD (3).

NMOSD typically affects females in their third and fourth decades (4), however, it can occur later in life (1, 4). Over the past few years, there has been increasing interest in the impact of onset age on the clinical features of NMOSD. Conventionally, patients with disease onset earlier than age 50 were classified as early-onset NMOSD (EO-NMOSD), whereas patients with disease onset at age 50 or older were classified as late-onset NMOSD (LO-NMOSD) (5, 6). Several studies have showed differences between LO-NMOSD and EO-NMOSD in aspects regarding attack types, anatomical preference, relapse rate, co-morbidities and prognosis. (6–8). However, some of these results were inconsistent due to limited sample size, mixed ethnicity, and incomplete clinical information. On the extreme end of the onset age, patients with disease onset at age 70 or older were defined as very-late-onset NMOSD (VLO-NMOSD). Though several cases reported that patients with VLO-NMOSD showed prominent extensive spinal cord lesions and worse prognosis (9–12), case series of VLO-NMOSD were rare (13, 14).

In this study, we aimed to investigate the clinical characteristics and outcomes in patients with different age of onset in a homogeneous cohort of the central-south Chinese population that included the largest cohort of LO-NMOSD with a detailed analysis of clinical and neuroimaging data. First, patients were divided into EO-NMOSD and LO-NMOSD using the age of onset at 50 years as a cut-off. To further address the effect of older age on NMOSD presentation, we reviewed our series of VLO-NMOSD and compared the clinical features and prognostic outcomes with that of relative late onset NMOSD (RLO-NMOSD, age of onset 50~69 years). Finally, we explored the correlations between age of onset and clinical characteristics and prognostic outcomes.

## Materials and methods

### Patient selection

Two-hundreds and ninety-eight NMOSD patients presented to the Department of Neurology and Geriatric Neurology, Xiangya Hospital in China between January 2016 to December 2021 were included in this study. Patients were enrolled if they met the following criteria: 1) fulfilled the diagnostic criteria recommended by the International Panel of NMOSD in 2015 (3); 2) followed for at least 6 months; 3) tested for serum AQP4-IgG using fixed cell-based indirect immunofluorescence staining.

### Data collection

We retrospectively reviewed the medical records and the information collected during follow-up, including sex, age, age of onset, disease duration, symptom at onset and relapses, number of relapses, the time interval from the first attack to the first relapse. Annualized relapse rate (ARR) and ARR excluding the first attack (ARR-1) were calculated based on above information. An attack/relapse was defined as the occurrence of new symptoms or worsening of existing symptoms that lasted for at least 24 hours and occurred at least 30 days after the previous attack. The first attack was defined as the index attack marking the disease onset. Based on clinical manifestations and neuroimaging findings obtained during the acute phase, the first attacks and relapses were classified into four different types: optic neuritis (ON), transverse myelitis (TM), brainstem or cerebral syndrome, and combined syndrome (defined as a combination of two or more above-mentioned core clinical characteristics).

Disability was evaluated by the Expanded Disability Status Scale (EDSS) score at the last follow-up (15). Motor dysfunction was defined as EDSS score ≥ 6, severe motor dysfunction was defined as EDSS score ≥ 8, and severe visual disability was defined as visual acuity ≤ 0.1.

Treatment during the acute phase of attacks (use of prednisone, plasmapheresis or intravenous immunoglobin) and maintenance therapy of immunosuppressive agents were also reviewed.

Laboratory results were collected including serostatus of AQP4-IgG, cerebrospinal fluid (CSF) analysis during the acute phase of attacks (cell count, protein level and oligoclonal bands), concomitant autoantibodies (antithyroglobulin antibody, antithyroid peroxidase antibody, antinuclear antibodies and anti-double stranded deoxyribonucleic acid antibody) and concomitant autoimmune diseases (Sjogren syndrome, autoimmune thyroid disease, systemic lupus erythematosus, and myasthenia gravis).

Brain MRIs were analyzed according to a prior review that summarized the neuroimaging features of NMOSD (16). The presence or absence of MRI lesions in the cerebrum, cerebellum, brainstem, and spinal cord were identified separately by two neurologists (ZW and YZ) based on reports by the neuroradiological department. In patients with myelitis episodes, the length of the spinal cord lesions was initially calculated independently by two neurologists (XL and YZ) and re-evaluated by an experienced neuroradiologist (HT). The length of spinal cord lesions was measured in terms of the number of vertebral segments.

### Statistical methods

Continuous data were presented as mean with standard deviation or median with inter-quartile range (IQR). Categorical variables were reported as absolute and relative frequencies. Continuous data were compared using Student’s t-test or Wilcoxon rank-sum test, whereas categorical data were compared using Chi-square or Fisher exact tests.The correlation between age of onset with clinical parameters was evaluated using Spearman’s or Pearson correlation analysis. Several baseline variables (onset age, sex, symptoms at first attack, abnormal CSF, serum status of AQP4-IgG, PE/IVIG during acute phase and maintenance treatment with IST) were assessed as possible predictors for development of disability using Cox proportional hazards regression. Baseline variables with a value of *p* < 0.1 in univariate Cox regression were included in the multivariate Cox regression model. The time to reach EDSS scores of 6.0 was estimated by the Kaplan-Meier method. Two-sided *p* values <0.05 were considered statistically significant. Statistical analyses were performed using SPSS (version 20.0, IBM Corp. Armonk, NY, USA).

## Results

### Demographic and clinical differences between EO-NMOSD and LO-NMOSD

Demographic and clinical data of the 298 patients, according to the age at disease onset, are summarized in **Table 1**. Overall, this study included 50 male and 248 female patients. The average age of disease onset was 46.2 ± 15.1 years, and the average duration of illness was 41.4 ± 35.7 months. Of the 298 patients, 164 (55.0%) were identified as EO-NMOSD, and 134 (45.0%) were identified as LO-NMOSD. The average onset age was 35.4 ± 10.0 years in EO-NMOSD and 59.5 ± 7.9 years in LO-NMOSD (*p* < 0.001). The disease duration was shorter in the LO-NMOSD group (34.3 ± 30.2 vs. 47.2 ± 38.8, *p* = 0.002).

**Table 1:** Comparison of demographic and clinical characteristics between EO-NMOSD and LO-NMOSD.

	NMOSD (n=298)	EO-NMOSD (n=164)	LO-NMOSD (n=134)	*p* Value
***Demographic***				
Female, n (%)	248 (83.2)	132 (80.5)	116 (86.6)	0.112
Age onset, year, Mean ± SD	46.2 ± 15.1	35.4 ± 10.0	59.5 ± 7.9	**<0.001**
Disease duration, month, Mean ± SD	41.4 ± 35.7	47.2 ± 38.8	34.3 ± 30.2	**0.002**
***Symptoms***				
Symptoms at onset, n (%)				
ON	88 (29.6)	57 (34.8)	31 (23.1)	**0.031**
TM	137 (46.0)	59 (36.0)	78 (58.2)	**0.002**
Brainstem/cerebral	40 (13.4)	30 (18.3)	10 (7.5)	**0.006**
Combined ^a^	33 (11.1)	18 (11.0)	15 (11.2)	>0.999
Symptoms involvement, n (%) ^b^				
ON	137 (46.0)	85 (51.8)	52 (38.8)	**0.027**
TM	250 (83.9)	132 (80.5)	118 (87.3)	0.083
Brainstem+Cerebral	127 (42.6)	78 (47.6)	49 (36.6)	0.061
ON+TM	113 (37.9)	65 (39.6)	48 (32.8)	0.549
***Relapses***				
Recurrent, n (%)	238 (79.9)	138 (84.2)	100 (74.6)	**0.044**
Total number of relapses, Mean ± SD	3.2 ± 2.0	3.6 ± 2.2	2.6 ± 1.5	**<0.001**
Time to first relapse, month, Mean ± SD	10.1 ± 10.0	9.8 ± 10.1	10.5 ± 10.0	0.602
ARR, Mean ± SD	1.3 ± 0.9	1.3 ± 0.8	1.3 ± 0.9	0.914
ARR-1, Mean ± SD	0.7 ± 0.6	0.7 ± 0.6	0.6 ± 0.5	**0.047**
***Disability***				
EDSS at last follow up, median (IQR)	2.5 (2.0-5.0)	2.5 (1.5 - 3.5)	3.5 (2.5 - 6.5)	**<0.001**
EDSS>6 at last follow up, n (%)	66 (22.2)	26 (15.9)	40 (29.9)	**0.005**
EDSS>8 at last follow up, n (%)	26 (8.7)	9 (5.5)	17 (12.7)	**0.038**
Visual acuity<0.1, n (%) ^c^	49 (16.4)	28 (17.1)	21 (15.7)	0.757
Death, n (%)	7 (2.3)	3 (1.8)	4 (3.0)	0.705
***Treatment***				
Acute phase treatment, n (%)				
Glucocorticoid	287 (96.3)	160 (97.6)	127 (94.8)	0.231
PE or IVIG	127 (42.6)	68 (41.5)	59 (44.0)	0.724
Chronic treatment of IST, n (%)	250 (83.9)	148 (90.2)	102 (76.1)	**0.001**
Mycophenolate Mofetil	201 (67.5)	113 (68.9)	88 (65.7)	0.619
Azathioprine	27 (9.1)	19 (11.6)	8 (6.0)	0.107
Tacrolimus	12 (4.0)	9 (5.5)	3 (2.2)	0.237
Rituximab	10 (3.4)	7 (4.3)	3 (2.2)	0.524
***Lab***				
AQP4-IgG positive, n (%)	247 (82.9)	134 (81.7)	113 (84.3)	0.643
Autoimmune antibodies, n (%)	103 (34.6)	51 (31.1)	52 (38.8)	0.180
Autoimmune diseases comorbidity, n (%)	55 (18.5)	32 (19.5)	23 (17.2)	0.654
Presence of OCB in CSF, n (%)	12 (6.8)	6 (6.5)	6 (7.1)	0.773
OCB unavailable, n (%)	121 (40.6)	71 (43.3)	50 (37.3)	0.343
Pleocytosis, n (%)	55 (18.5)	29 (17.7)	26 (19.4)	0.765
Increased CSF protein level, n (%)	72 (24.2)	33 (20.1)	39 (29.1)	0.078
***MRI***				
Length of Spinal lesions^d^				
Detailed data available, n (%) ^e^	243 (81.5)	130 (79.3)	113 (84.3)	0.366
First attack, median (IQR)	5.0 (3.0-8.0)	5.0 (3.0-7.0)	6.0 (3.0-9.0)	**0.002**
At last follow up, median (IQR)	6.0 (4.0-9.0)	5.5 (3.75-8.0)	6.0 (4.0-11.0)	**0.011**
Brain lesions, n (%)				
NMOSD typical lesion	132 (44.3)	82 (50.0)	50 (37.3)	**0.035**
Area postrema	35 (11.7)	26 (15.9)	9 (6.7)	**0.018**
Brainstem/cerebellum	73 (24.5)	46 (28.1)	27 (20.2)	0.137
Adjacent to 3^rd^ ventricle	41 (13.8)	26 (15.9)	15 (11.2)	0.311
Surrounding lateral ventricles	41 (13.8)	22 (13.4)	19 (14.2)	0.867
Pyramidal tracts involvement	23 (7.7)	16 (9.8)	7 (5.2)	0.191
Extensive hemispheric lesions	11 (3.7)	7 (4.3)	4 (3.0)	0.760

NMOSD, neuromyelitis optica spectrum disorder; EO, early-onset; LO-NMOSD, late-onset; SD, stand deviation; IQR, inter-quartile range; ON, optic neuritis; TM, transverse myelitis; ARR, annualized relapse rate; ARR-1, ARR excluding the first attack; EDSS, Expanded Disability Status Scale; PE, plasma exchange; IVIG, intravenous immunoglobin; IST, immunosuppressive therapy; AQP4-IgG, aquaporin-4 immunoglobin G; CSF, cerebrospinal fluid; OCB, oligoclonal band.

a Combined syndrome: defined as a combination of two or more core clinical characteristics.

b Proportions of symptoms involved during the entire disease duration.

c For the comparison of visual acuity, only those patients who had at least 1 optic neuritis attack were considered.

d Length of the spinal cord lesions were measured in terms of the number of vertebral segments.

e For the comparison of length of spinal cord lesions, only those patients who had at least 1 transverse myelitis attack were considered.

p values <0.05 were bolded.

As for the first attack, LO-NMOSD patients showed more frequent TM (58.2% vs. 36.0%, *p* = 0.007), while less frequent ON (23.1% vs. 34.8%, *p* = 0.031) and brain/brainstem attacks (7.5% vs. 18.3%, *p* = 0.006), compared to EO-NMOSD. During the entire disease course, the tendencies were similar to that of the initial attack, but the difference was insignificant (*p*=0.091, 0.083, and 0.061, respectively).

The recurrent course was less frequent in LO-NMOSD patients compared EO-NMOSD (84.2% vs. 74.6%, *p* = 0.044). While the time between the first attack to the first relapse and ARR was not significantly different between the two groups, EO-NMOSD patients showed higher ARR-1 (0.7 ± 0.6 vs. 0.6 ± 0.5, *p* = 0.047) compared to LO-NMOSD.

At the last follow up, LO-NMOSD showed higher EDSS score (3.5 vs. 2.5, *p* < 0.001), higher proportion of motor dysfunction (EDSS≥6) (29.9% vs. 15.9%, *p* = 0.005) and severe motor dysfunction (EDSS≥8) (12.7% vs 5.5%, *p* = 0.038). The proportion of visual disability and death rate was similar between the two groups.

The acute treatment with glucocorticoid and plasma exchange (PE) or intravenous immunoglobin (IVIG) was similar between the two groups. For relapse prevention, the proportion of patients taking oral prednisone was similar between EO-NMOSD and LO-NMOSD, while the proportion of patients taking immunosuppressive agents was lower in the LO-NMOSD group than that in EO-NMOSD (76.1% vs. 90.2%, *p* = 0.001).

The proportions of patients with AQP-4 IgG were similar between EO-NMOSD and LO-NMOSD (81.7% vs 84.3%, *p* = 0.55). Other autoimmune antibodies, concurrent autoimmune diseases, increased CSF cell count or protein, and the presence of CSF OCB was not significantly different between the two groups.

LO-NMOSD patients showed longer vertebral segments of the spinal lesion at the first attack (6.0 vs 5.0, *p* = 0.002), and at the last follow-up (6.0 vs 5.5, *p* = 0.011), compared to EO-NMOSD patients. Typical NMOSD brain lesions were less frequently observed in the LO-NMOSD group than in the EO-NMOSD group (37.3% vs. 50.0%, *p* = 0.035). In particular, area postrema (AP) lesions were less frequently observed in LO-NMOSD group (6.7% vs. 15.9%, *p* = 0.018).

### Demographic and clinical differences between RLO-NMOSD and VLO-NMOSD

We divided LO-NMOSD into 2 subgroups: RLO-NMOSD and VLO-NMOSD using the age of onset at 70 years as a cut-off. Demographic and clinical features are summarized in **Table 2**. Of the 134 patients with LO-NMOSD, 15 (11.2%) were VLO-NMOSD patients. The average onset age was 57.6 ± 6.0 years in RLO-NMOSD, and 74.5 ± 3.3 years in VLO-NMOSD. The VLO-NMOSD group showed a lower proportion of female patients than RLO-NMOSD (66.7% vs 89.1%, *p* = 0.032). Though the disease duration was shorter in VLO-NMOSD (36.1 ± 31.4 vs. 19.7 ± 9.4, *p* = 0.047), the parameters regarding relapses (including total relapsing times, time to first relapse, ARR, and ARR-1) were not significantly different between two groups.

**Table 2:** Comparison of demographic and clinical characteristics between RLO-NMOSD and VLO-NMOSD.

	RLO-NMOSD (n=119)	VLO-NMOSD (n=15)	*p* Value
**Demographic**			
Female, n (%)	106 (89.1)	10 (66.7)	**0.032**
Age onset, year, Mean ± SD	57.6 ± 6.0	74.5 ± 3.3	**0.000**
Disease duration, month, Mean ± SD	36.1 ± 31.4	19.7 ± 9.4	**0.047**
**Symptoms**			
Symptoms at onset, n (%)			
ON	30 (25.2)	1 (6.7)	0.190
TM	65 (54.6)	13 (86.7)	**0.024**
Brainstem/cerebral	10 (8.4)	0 (0.0)	0.602
Combined ^a^	14 (11.8)	1 (6.7)	>0.999
Symptoms involvement, n (%) ^b^			
ON	49 (41.2)	3 (20.0)	0.161
TM	104 (87.4)	14 (93.3)	>0.999
Brainstem+Cerebral	45 (37.8)	4 (26.7)	0.571
ON+TM ^a^	45 (37.8)	3 (20.0)	0.255
**Relapses**			
Recurrent, n (%)	90 (75.6)	10 (66.7)	0.530
Total number of relapses, Mean ± SD	2.6 ± 1.5	2.1 ± 1.0	0.159
Time to first relapse, month, Mean ± SD	10.7 ± 10.4	8.2 ± 5.0	0.452
ARR, Mean ± SD	1.3± 0.9	1.5 ± 1.0	0.295
ARR-1, Mean ± SD	0.6 ± 0.5	0.7± 0.6	0.431
**Disability**			
EDSS at last follow up, median (IQR)	3 (2.0-6.5)	6 (3.5-8.0)	**0.026**
EDSS>6 at last follow up, n (%)	32 (26.9)	8 (53.3)	0.068
EDSS>8 at last follow up, n (%)	13 (10.9)	4 (26.7)	0.100
Visual acuity<0.1, n (%) ^c^	19 (16.0)	2 (13.3)	>0.999
Death, n (%)	3 (2.5)	1 (6.7)	0.382
**Treatment**			
Acute phase treatment, n (%)			
Glucocorticoid	114 (96.0)	13 (86.7)	0.177
PE or IVIG	49 (41.2)	10 (66.7)	0.096
Chronic treatment of IST, n (%)	95 (79.8)	7 (46.7)	**0.009**
Mycophenolate Mofetil	81 (68.1)	7 (46.7)	0.147
Azathioprine	8 (6.7)	0 (0.0)	0.597
Tacrolimus	3 (2.5)	0 (0.0)	>0.999
Rituximab	3 (2.5)	0 (0.0)	>0.999
**Lab**			
AQP4-IgG positive, n (%)	100 (84.0)	13 (86.7)	>0.999
Autoimmune antibodies, n (%)	44 (37.0)	8 (53.3)	0.265
Autoimmune diseases comorbidity, n (%)	22 (18.5)	1 (6.7)	0.467
Presence of OCB in CSF, n (%)	5 (6.9)	1 (6.7)	0.517
OCB unavailable, n (%)	46 (38.7)	4 (26.7)	0.413
Pleocytosis, n (%)	23 (19.3)	3 (20.0)	0.736
Increased CSF protein level, n (%)	32 (26.9)	7 (46.7)	0.135
**MRI**			
Length of Spinal lesions^d^			
Detailed data available, n (%) ^e^	98 (82.4)	14 (93.3)	0.464
First attack, median (IQR)	5.75 (3.0-8.25)	9.0 (7.0-9.0)	**0.015**
At last follow up, median (IQR)	6.0 (4.0-10.0)	10.5 (7.5-14.25)	**0.007**
Brain lesions, n (%)			
NMOSD typical lesion	47 (39.5)	3 (20.0)	0.167
Area postrema	8 (6.7)	1 (6.7)	>0.999
Brainstem/cerebellum	26 (21.9)	1 (6.7)	0.303
Adjacent to 3^rd^ ventricle	14 (11.8)	1 (6.7)	>0.999
Surrounding lateral ventricles	16 (13.5)	3 (20.0)	0.447
Pyramidal tracts involvement	7 (5.9)	0 (0.0)	>0.999
Extensive hemispheric lesions	4 (3.4)	0 (0.0)	>0.999

NMOSD, neuromyelitis optica spectrum disorder; RLO, very late onset; VLO, relative late onset; SD, stand deviation; IQR, inter-quartile range; ON, optic neuritis; TM, transverse myelitis; ARR, annualized relapse rate; ARR-1, ARR excluding the first attack; EDSS, Expanded Disability Status Scale; PE, plasma exchange; IVIG, intravenous immunoglobin; IST, immunosuppressive therapy; AQP4-IgG, aquaporin-4 immunoglobin G; CSF, cerebrospinal fluid; OCB, oligoclonal band.

a Combined: defined as a combination of two or more core clinical characteristics.

b Proportions of symptoms involved during the entire disease duration.

c For the comparison of visual acuity, only those patients who had at least 1 optic neuritis attack were considered.

d Length of the spinal cord lesions were measured in terms of the number of vertebral segments.

e For the comparison of length of spinal cord lesions, only those patients who had at least 1 transverse myelitis attack were considered.

p values <0.05 were bolded.

As for the first attack, VLO-NMOSD patients showed more frequent TM compared to RLO-NMOSD (86.7% vs. 56.6%, *p* = 0.0242). The involvement of optic neuritis and brainstem/cerebral syndrome were similar between the two groups.

VLO-NMOSD patients showed more severe disability compared to RLO-NMOSD, as measured by EDSS score at last follow-up (3.0 vs. 5.5, *p* = 0.026). Severe visual disability and death was not significantly different between two groups.

Regarding neuroimaging characteristics, VLO-NMOSD patients showed longer segments of spinal lesions compared to RLO-NMOSD at first attack (9.0 vs. 5.75, *p* = 0.015), and last follow-up (10.5 vs 6.0, *p* = 0.007). The distribution of brain lesions didn’t show differences between the two groups.

### Demographic and clinical differences sorted by onset age in patients with AQP4-IgG

To exclude the effect of AQP4 antibody-negative patients, we compared characteristics between EO-NMOSD and LO-NMOSD in patients with AQP4-IgG (**Supplementary Table 1**), as well as that between RLO-NMOSD and LO-NMOSD in patients with AQP4-IgG (**Supplementary Table 2**). Most of the differences identified between EO-NMOSD and LO-NMOSD patients in the entire cohort were replicated in patients restricted to AQP4-IgG positive patients, except for the proportions of patients reached EDSS 8 at the last follow-up (*p*=0.03 in entire corhort and *p*=0.065 in AQP4-IgG positive cohort), which may be attributed to decreased sample size and low incidence of patients reaching EDSS score of 8.

### Correlations of onset age with clinical, neuroimaging, and prognostic parameters

**Figure 1:**
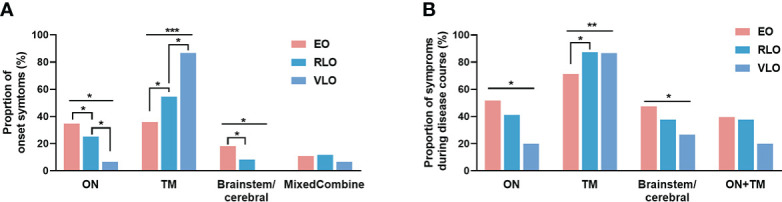
Proportion of symptoms by age groups of NMOSD patients. **(A)** Symptoms at onset: at disease onset, the proportion of patients with optic neuritis and brainstem/cerebral symptoms decreased with age (*p* = 0.030 and *p* = 0.016, respectively); the proportion of patients with transverse myelitis increased with age (*p* < 0.001). **(B)** Symptoms during entire disease course: the involvement of optic neuritis and brain/cerebellum symptoms decreased with onset age (*p* = 0.024 and *p* = 0.038, respectively); the involvement of transverse myelitis increased with onset age (*p* = 0.004). NMOSD, Neuromyelitis optica spectrum disorder; ON, optic neuritis; TM: transverse myelitis; Combined:,any combination of more than one clinical presentation in NMOSD, EO: early-onset NMOSD; RLO, relative-late-onset NMOSD; VLO, very-late-onset NMOSD. *p < 0.05; **p < 0.01; ***p < 0.001.

With increasing age of disease onset, the proportion of patients with optic neuritis and brain/cerebellum symptoms as initial attack decreased (*p* = 0.030 and *p* = 0.016, respectively), while the proportion of patients with transverse myelitis increased (*p* < 0.001) (**Figure 1A**). During the entire disease course, the involvement of optic neuritis and brain/cerebellum symptoms decreased with onset age (*p* = 0.024 and *p* = 0.038, respectively), while the involvement of transverse myelitis increased with onset age (*p* = 0.004) (**Figure 1B**).

**Figure 2:**
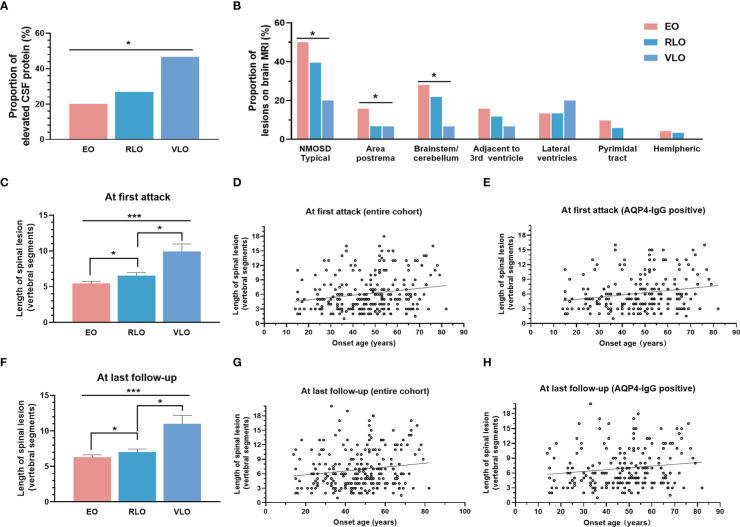
Correlation of onset age with laboratory and neuroimaging characteristics. **(A)** Proportion of elevated CSF protein level increased with groups by age of onset (*p* = 0.022). **(B)** Brain lesion distribution on MRI: patients with older onset age showed less typical NMOSD brain lesions (*p* = 0.032), especially less lesions in area postrema (*p* = 0.023) and brainstem/cerebellum (*p* = 0.049); (c-e): Length of spinal cord lesion at first onset: length of spinal cord lesion increased with groups by age of onset **(C)** (*p* < 0.001), positively correlated with age of onset in entire cohort **(D)** (*r* = 0.1789, *p* = 0.005), and in AQP4-IgG positive NMOSD patients **(E)** (*r* = 0.2021, *p* = 0.004); **(F-H)** Length of spinal cord lesion at last follow up: length of spinal cord lesion increased with groups by age of onset **(F)** (*p* < 0.001), positively correlated with age of onset in entire cohort **(G)** (*r* = 0.1512, *p* = 0.019), and in AQP4-IgG positive NMOSD patients **(H)** (*r* = 0.1453, *p* = 0.040). NMOSD, Neuromyelitis optica spectrum disorder; CSF, cerebrospinal fluid; AQP4, aquaporin-4; EO, early-onset NMOSD, RLO: relative-late-onset NMOSD, VLO: very-late-onset NMOSD. *p < 0.05; ***p < 0.001.

The proportion of patients with elevated CSF protein levels increased with onset age (*p* = 0.022) (**Figure 2A**). Patients with older age of onset exhibited less typical NMOSD brain lesions (*p* = 0.032), especially less lesions in area postrema (*p* = 0.023) and brainstem/cerebellum (*p* = 0.049) (**Figure 2B**). The length of spinal cord lesion measured by vertebrate segments increased with age of onset at first attack and at last follow-up (*p* < 0.001) (**Figures 2C, F**). We even observe a positive correlation between the onset age and length of spinal cord at first attack (*r* = 0.1789, *p* = 0.005) (**Figure 2D**), as well as at last follow up (*r* = 0.1512, *p* = 0.019) (**Figure 2G**). The correlation was also significant in AQP4-IgG positive patients (*r* = 0.2021, *p*=0.004 and *r* = 0.1453, *p* = 0.040, respectively) (**Figures 2E, H**).

**Figure 3:**
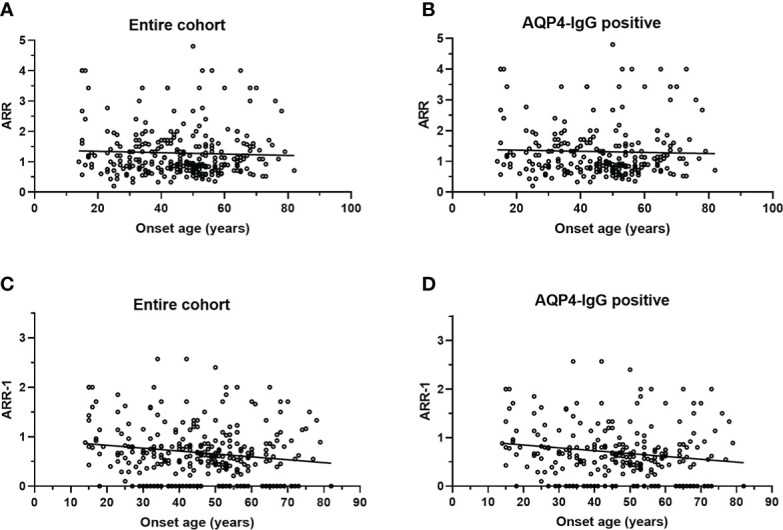
Scatterplot for correlation of onset age with ARR and ARR-1. **(A)** ARR was not significantly correlated with onset age in entire cohort (*p* = 0.483); **(B)** ARR was not significantly correlated with onset age in patients with AQP4-IgG (*p* = 0.613); **(C)** ARR-1 inversely correlated with onset age (*r* = −0.1552, *p* = 0.007) in entire cohort; **(D)** ARR-1 inversely correlated with onset age (*r* = −0.1568, *p* = 0.013) in patients with AQP4-IgG. NMOSD, Neuromyelitis optica spectrum disorder; ARR, annualized relapse rate; ARR-1: ARR, excluding the first attack; AQP4, aquaporin-4; EO, early-onset NMOSD, RLO: relative-late-onset NMOSD, VLO: very-late-onset NMOSD.

No significant correlation was observed between age at onset with ARR in the entire cohort (**Figure 3A**) or AQP4-IgG positive patients (**Figure 3B**). There is a negative correlation between age at onset with ARR-1 in the entire cohort (*r* =−0.1552, *p* = 0.007) (**Figure 3C**) and in AQP4-IgG positive patients (*r* =−0.1568, *p* = 0.013) (**Figure 3D**).

**Figure 4:**
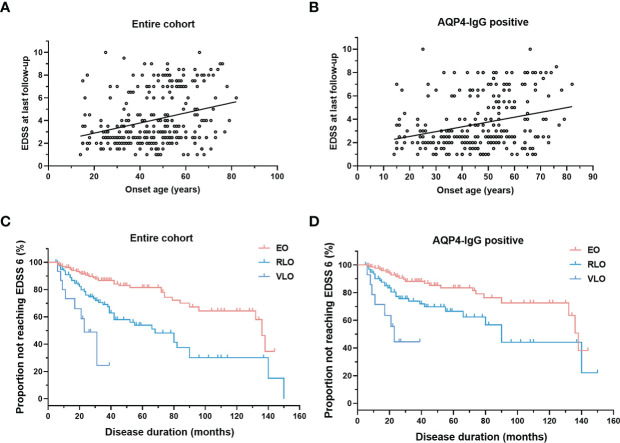
Correlation of onset age with EDSS. **(A, B)** Scatterplot for correlation of onset age with EDSS: EDSS positively correlated with onset age in entire cohort (*r* = 0.2897, *p* < 0.0001) and in patients with AQP4-IgG (*r* = 0.2834, *p* < 0.0001); **(C, D)** Survival curve of patients not reaching motor disability (EDSS score of 6): patients with older onset age reach EDSS score of 6.0 sooner in entire cohort (Kaplan-Meier method, *p* < 0.001), and in patients with AQP4-IgG (Kaplan-Meier method, *p* < 0.001). NMOSD, Neuromyelitis optica spectrum disorder; EDSS, Expanded Disability Status Scale; AQP4, aquaporin-4; EO, early-onset NMOSD, RLO: relative-late-onset NMOSD, VLO: very-late-onset NMOSD.

There is a highly significant positive correlation between age of onset with EDSS score at the last follow-up (**Figures 4A, B**), irrespective of AQP4-IgG serostatus (*p* < 0.001). Univariate and multivariate analysis to predict disability (EDSS ≥6 and EDSS≥8) were performed using Cox proportional hazards regression models (**Table 3**). In univariate Cox regression, we found that for every 10-year increase in age at disease onset, the risk of motor disability (EDSS≥6) increased by 59% (hazard ratio [HR] 1.59, 95% CI 1.31-1.94, *p* < 0.001). Other significant predictors identified in the univariate Cox proportional hazards regression were ON as the initial attack (hazard ratio [HR] 0.45, 95% CI 0.24 - 0.82, *p =* 0.009), abnormal CSF (hazard ratio [HR] 2.24, 95% CI 1.35 - 3.70, *p =* 0.002) and maintenance treatment with IST (hazard ratio [HR] 0.52, 95% CI 0.29 - 0.93, *p* = 0.028). The contribution of age at onset (hazard ratio [HR] 1.47, 95% CI 1.20 - 1.82, *p* < 0.001), ON as the first attack (hazard ratio [HR] 0.50, 95% CI 0.19 - 0.94, *p =* 0.035), and abnormal CSF (hazard ratio [HR] 2.26, 95% CI 1.34 - 3.83, *p =* 0.002) were still significant in the multivariate Cox regression model predicting EDSS≥6 at the last follow-up. In the entire cohort, patients with older onset age took a shorter median time to reach EDSS ≥6 at the last follow up (*p* < 0.001), irrespective of AQP4-IgG serostatus (**Figures 4C, D**).

**Table 3:** Univariate and multivariate Cox proportional hazard regression analysis for disability.

Baseline Variables	EDSS≥6				EDSS≥8			
	Univariate		Multivariate		Univariate		Multivariate	
	HR (95% CI)	*p* value	HR (95% CI)	*p* value	HR (95% CI)	*p* value	HR (95% CI)	*p* value
Onset age	1.59 (1.31 - 1.94)	**<0.001**	1.47 (1.20- 1.88)	**<0.001**	2.05 (1.46 - 2.89)	**<0.001**	1.74 (1.25 - 2.47)	**0.002**
Female	1.02 (0.52 - 1.99)	0.964	/	/	0.73 (0.28 - 1.94)	0.526	/	/
TM as first attack	1.58 (0.97 - 2.57)	0.068	1.04 (0.54 - 1.93)	0.906	1.39 (0.64 - 3.02)	0.403	/	/
ON as first attack	0.45 (0.24 - 0.82)	**0.009**	0.50 (0.19 - 0.94)	**0.035**	0.35 (0.12- 1.02)	0.055	0.38 (0.12 - 1.18)	0.093
Brainstem/Cerebral as first attack	1.14 (0.54 - 2.40)	0.725	/	/	0.55 (0.23 - 1.31)	0.179	/	/
Abnormal CSF	2.24 (1.35 - 3.70)	**0.002**	2.26 (1.34 - 3.83)	**0.002**	3.20 (1.43 - 7.18)	**0.005**	2.95 (1.29 - 6.74)	**0.010**
AQP4-IgG positive	1.03 (0.53 - 2.04)	0.923	/	/	1.32 (0.49 - 3.54)	0.581	/	/
PE/IVIG during acute phase	1.58 (0.98 - 2.56)	0.063	1.43 (0.86 - 2.40)	0.172	0.68 (0.32 - 1.47)	0.330	/	/
Maintenance treatment of IST	0.52 (0.28 - 0.93)	0.028	0.72 (0.37 - 1.36)	0.302	0.26 (0.11 - 0.61)	**0.002**	0.43 (0.17 - 1.05)	0.063

EDSS, Expanded Disability Status Scale; HR, hazard ratio; CI, confidence interval; TM, transverse myelitis; ON, optic neuritis; CSF, cerebrospinal fluid; AQP4-IgG, aquaporin-4 immunoglobin G; PE, plasma exchange; IVIG, intravenous immunogloblin; IST, immunosuppressive therapy.

p values <0.05 were bolded.

## Discussion

This is a retrospective study focused on onset-age-dependent clinical characteristics of NMOSD patients in a Chinese cohort including 298 patients. The proportion of LO-NMOSD included in our study (44.9%) is higher than that in previous ones, ranging from 17.1% to 30.6% (6–8, 17). The possible reason is that we included a substantial number of patients from the National Clinical Research Center for Geriatric Disorders of Xiangya Hospital, therefore, we cannot infer that late-onset NMOSD is more common in our population as compared to other populations.

We observed that LO-NMOSD patients developed transverse myelitis more frequently at disease onset, and the prevalence of transverse myelitis as initial attack increases with age of onset. These results were similar to most previous studies in patients from Caucasian, Asian (7, 8), European (6), and Latin American (17) populations. We also observed that LO-NMOSD patients developed optic neuritis less frequently at disease onset. The prevalence of optic neuritis syndromes as initial attack decreases with age of onset. Similar results have been reported in several previous studies (8, 18). What’s more, we found that patients with LO-NMOSD had a lower risk of brain/brainstem syndromes at disease onset as well as less NMOSD typical brain lesions on MRI. These above results indicated that there are differences in anatomical susceptibility in patients with different onset age, that the optic nerve and brain were more susceptible in young patients, while the spinal cord was more vulnerable in aged patients. Consistent with this pattern, studies have showed that pediatric NMOSD patients had a higher risk of visual disability and brain lesions compared to adult patients (18–20). Similar anatomical preference has been observed in multiple sclerosis (MS), that early-onset MS patients showed more prominent visual disturbance while milder spinal cord involvement compared to late-onset MS (21, 22). It is possible that this onset-age-dependent anatomical susceptibility may be non-disease-specific and might be related to different vulnerability of blood-brain barrier and blood-spinal cord barrier during aging. In patients with myelin oligodendrocyte glycoprotein (MOG) antibody-associated demyelination (MOGAD), studies have shown that adults patients with MOGAD showed less frequent encephalitis (ADEM phenotype) while more frequent myelitis and optic neuritis compared to pediatric patients with MOGAD (23, 24). Though anatomical preference in adult patients grouped by ages or onset ages has not been well described yet, the preferential impairment of ON in adult patients over pediatric patients in MOGAD is different from what we observed in NMOSD.

In terms of relapsing, the relapsing frequency measured by ARR-1 was lower in LO-NMOSD compared to EO-NMOSD. This result is consistent with mots previous studies (7, 8, 25, 26). In addition, we observed an inverse correlation between ARR-1 and age of onset, suggesting a lower relapsing rate in patients with older onset age. On the other hand, several studies showed similar relapsing rate between LO-NMOSD and EO-NMOSD measured by ARR (6, 8). It is proposed that ARR-1 may be more accurate to avoid inappropriately high ARR values in patients with disease duration less than 1 year (5, 8, 27, 28). In line with this, ARR was not significantly correlated with onset age in our study.

Worse prognosis and severer motor disability in LO-NMOSD have been demonstrated in several previous studies (5–8, 17, 25, 28, 29), as well as in the present study: the EDSS score at last follow-up increases with onset age; older age at disease onset predict motor disability and severe disability at last follow-up; patients with older age of onset progress to motor disability more quickly. The reasons for worse prognosis in LO-NMOSD were unclear yet. It is noteworthy that the frequency of maintenance therapy with IST was lower in patients with LO-NMOSD than patients with EO-NMOSD, however, the protective effect of IST from development of disability were not confidently confirmed in our study. Because patients with NMOSD rarely present with a progressive clinical course, disability is generally thought to accumulate from frequent relapses or poor recovery after severe episodes. In our group, LO-NMOSD patients showed severer disability with shorter disease duration, less relapsing times, and lower relapsing rate (measured by ARR-1) compared to EO-NMOSD, suggesting that the disability was less likely accumulated through frequent relapses. A recent study demonstrated that age and a worse recovery from the first attack were the main predictor factors of disability in NMOSD (6), suggesting that impaired reparation mechanism in aged patients may be the main reason leading to worse prognosis. In addition, patients with transverse myelitis at the time of the first episode have been shown to predict severe disability in NMOSD (25). Thus, the higher proportion of transverse myelitis at disease onset and the impaired restorative capacity may partly explain the poorer prognosis of LO-NMOSD.

The results of visual disability were inconsistent among studies. Several studies found that LO-NMOSD patients were more susceptible to visual disability (8, 28), while others come to the opposite conclusion (7, 25, 30). Especially, in a large cohort from the United Kingdom and Japan, results suggested that the likelihood of developing visual disability decreases with the age of onset (25). However, we didn’t observe significant differences in visual disability among patients sorted by onset age in present study.

There have been anecdotal cases demonstrating extensive longitude spinal cord lesions in VLO-NMOSD patients. In 16 previously reported VLO-NMOSD cases with transverse myelitis, more than half of the patients developed spinal cord lesions exceeding 8 vertebral segments (9–12, 31–38). Case series of VLO-NMOSD is rare (13, 14). One case series from the Japanese population including eight VLO-NMOSD patients showed that the average length of spinal lesions of VLO-NMOSD was ten vertebral segments, which is significantly longer than that of LO- and EO-NMOSD patients (14). Above observations suggested that patients with older onset age may present with longer segments of spinal cord lesions. There have been only a few studies focused on the differences in length of spinal lesions between NMOSD patients with different onset age (5, 7, 30, 39). In this study, we found that VLO-NMOSD patients showed longer segments of spinal lesion compared to patients with RLO-NMOSD, both at first attack and at last follow-up. Also, patients with LO-NMOSD showed longer segments of spinal lesion compared to patients with EO-NMOSD, both at first attack and at the last follow-up. Moreover, the length of spinal cord lesions positively correlated with onset age. These results suggested that patients with older onset age were more likely to develop longer segments of spinal cord lesions. In addition to higher occurrence of transverse myelitis at disease onset and impaired reparation capacity, longer segments of spinal cord lesions may also contribute to poor motor prognosis in LO-NMOSD.

The reason why LO-NMOSD patients exhibited longer segments of spinal cord lesions was unknown. In present study, although utilization of IST was less frequent in patients with RLO- and VLO-NMOSD, the total relapsing times and relapsing rate measured by ARR-1 were decreased compared to that in EO-NMOSD, suggesting that extensive spinal cord impairment was not caused by more relapses or longer disease duration. The above results suggested that in patients with aged NMOSD patients, neurological disability may be attributed to more aggressive and extensive inflammation during limited relapses.

We reviewed 16 previously reported VLO-NMOSD cases and found that only 2 cases showed negative CSF analysis during the attack (9–12, 31–38). Pleocytosis or increased protein CSF was detected in 87.5% (14/16) patients. In our study, there is a tendency that cerebrospinal fluid protein levels during the acute phase of attacks increased with the age of disease onset, reflecting a more aggressive inflammatory reaction during the acute phase of attacks in aged NMOSD. However, this tendency didn’t reach statistical significance between LO-NMOSD and EO-NMOSD, and between VLO-NMOSD and RLO-NMOSD, we suspected that limited sample size and different timing of lumber punctures may be responsible, so this need to be confirmed in large cohorts with consistent timing of CSF analysis.

There are several limitations of our study. It is a retrospective study, the recalling biases were inevitable. Several factors such as differences in disease duration and timing of the neuroimaging evaluation or CSF analysis may confound the conclusion. In addition, we only focused on AQP4-IgG serostatus, and the anti-MOG antibody was not tested. The sample size, especially the VLO-NMOSD group was limited.

## Conclusion

In conclusion, this study demonstrated that patients with older age of onset exhibit more frequent transverse myelitis, less frequent optic neuritis, fewer NMOSD-typical brain lesions on MRI, longer segments of spinal cord lesions, less frequent relapses, more aggressive disease progress and severer disability. These results suggested that age of disease onset correlates with clinical characteristics and prognostic outcomes in patients with NMOSD. These findings need to be further confirmed in prospective studies with larger sample size and strictly controlled multiple variables.

## Data availability statement

The datasets presented in this study can be found in online repositories. The names of the repository/repositories and accession number(s) can be found below: https://doi.org/10.6084/m9.figshare.21116827.

## Ethics statement

The studies involving human participants were reviewed and approved by Ethics Committee of Xiangya Hospital, Central South University. The patients/participants provided their written informed consent to participate in this study.

## Author contributions

YH, YZ and LZ designed the research. YH, QS, LY, YT, ML, NX, YZ and FY collected clinical data. HT provided and evaluated neuroimages information. XL, NX, ZW and YH performed the statistical analysis and visualized the data. YH and ZW wrote the manuscript. QS, HX and YZ supervised the study and revised the manuscript. YH and YZ provided funding support. All authors contributed to the article and approved the submitted version.
