# Supplementary material for: Age of onset correlates with clinical characteristics and prognostic outcomes in neuromyelitis optica spectrum disorder
Source: Front Immunol. 2022 Dec 8;13:1056944. doi: 10.3389/fimmu.2022.1056944 (PMC9772011; doi:10.3389/fimmu.2022.1056944)
Supplement: Supplementary file 1 [file Table_1.docx]

Table1 Comparison of demographic and clinical characteristics between EO-NMOSD and LO-NMOSD

in patients with AQP4-IgG

|  | **AQP4-IgG (+)**  **(n=247)** | **EO-NMOSD**  **(n=134)** | **LO-NMOSD**  **(n= 113)** | ***p* Value** |  |
| --- | --- | --- | --- | --- | --- |
| ***Demographic*** |  |  |  |  |  |
| Female, n (%) | 211 (85.4) | 110 (82.1) | 101 (89.4) | 0.147 |  |
| Age onset, year, Mean ± SD | 46.4 ± 15.6 | 34.9 ±10.4 | 59.8 ±7.9 | **0.001** |  |
| Disease duration, month, Mean ± SD | 41.6 ± 35.8 | 48.2 ± 38.6 | 33.9 ± 30.7 | **0.001** |  |
| ***Symtoms*** |  |  |  |  |  |
| Symtoms at onset, n (%) |  |  |  |  |  |
| ON | 76 (30.8) | 49 (36.6) | 27 (24.0) | **0.038** |  |
| TM | 113 (45.7) | 46 (34.3) | 67 (59.3) | **<0.001** |  |
| Brainstem+Cerebral | 34 (12.1) | 25 (18.7) | 9 (8.0) | **0.016** |  |
| Combined ^a^ | 24 (9.7) | 14 (10.4) | 10 (8.8) | 0.674 |  |
| Symptoms involvement, n (%) ^b^ |  |  |  |  |  |
| ON | 113 (45.7) | 72 (53.7) | 41 (36.3) | **0.007** |  |
| TM | 191 (77.3) | 91 (67.9) | 100 (88.4) | **<0.001** |  |
| Brainstem+Cerebral | *98* (39.7) | 59 (44.0) | 39 (34.5) | 0.120 |  |
| ON+TM | 92 (37.2) | 54 (40.4) | 37 (32.7) | 0.236 |  |
| ***Relapses*** |  |  |  |  |  |
| Recurrent, n (%) | 199 (80.1) | 117 (87.3) | 82 (72.6) | **0.006** |  |
| Total number of relapses, Mean ± SD | 3.2 ± 1.9 | 3.7 ± 2.1 | 2.45 ± 1.4 | **<0.001** |  |
| Time to first relapse, month, Mean ± SD | 10.3 ± 10.2 | 9.9 ± 10.3 | 10.8 ± 10.1 | 0.521 |  |
| ARR, Mean ± SD | 1.3 ± 0.9 | 1.319 ± 0.8 | 1.285 ± 0.9 | 0.757 |  |
| ARR-1, Mean ± SD | 0.7 ± 0.6 | 0.8 ± 0.6 | 0.6 ± 0.5 | **0.012** |  |
| ***Disability*** |  |  |  |  |  |
| EDSS at last follow up, median (IQR) | 2.5 (2.0 - 5.5) | 2.5 (1.5-3.5) | 4.0 (2.5-6.5) | **<0.001** |  |
| EDSS＞6 at last follow up, n (%) | 56 (22.7) | 22 (16.4) | 34 (30.1) | **0.014** |  |
| EDSS＞8 at last follow up, n (%) | 21 (8.5) | 7 (5.2) | 14 (12.4) | 0.065 |  |
| Visual acuity<0.1, n (%) ^c^ | 39 (15.8) | 20 (14.9) | 19 (16.8) | 0.729 |  |
| Death, n (%) | 5 (2.0) | 2 (1.5) | 3 (2.6) | 0.664 |  |
| ***Treatment*** |  |  |  |  |  |
| Acute phase treatment, n (%) |  |  |  |  |  |
| Glucocorticoid | 243 (98.4) | 132 (98.5) | 111 (98.2) | >0.999 |  |
| PE or IVIG | 105 (42.5) | 57 (42.5) | 48 (42.5) | >0.999 |  |
| Chronic treatment of IST, n (%) | 209 (84.6) | 126 (94.0) | 83 (73.5) | **<0.001** |  |
| Mycophenolate Mofetil | 165 (66.8) | 95 (70.1) | 70 (61.9) | 0.175 |  |
| Azathioprine | 24 (9.7) | 16 (11.9) | 8 (7.1) | 0.281 |  |
| Tacrolimus | 11 (4.5) | 9 (6.7) | 2 (1.8) | 0.070 |  |
| Rituximab | 9 (3.6) | 6 (4.5) | 3 (2.6) | 0.514 |  |
| ***Lab*** |  |  |  |  |  |
| Autoimmune antibodies, n (%) | 96 (38.9) | 49 (36.6) | 47 (41.6) | 0.435 |  |
| Autoimmune diseases comorbidity, n (%) | 56 (22.7) | 33 (24.6) | 23 (20.4) | 0.449 |  |
| Presence of OCB in CSF, n (%) | 10 (6.7) | 5 (6.4) | 5 (7.0) | >0.999 |  |
| OCB unavailable, n (%) | 98 (39.7) | 56 (41.8) | 42 (37.2) | 0.515 |  |
| Pleocytosis, n (%) | 47 (19.0) | 21 (15.6) | 26 (23.0) | 0.148 |  |
| Increased CSF protein level, n (%) | 53 (21.5) | 20 (14.9) | 33 (29.2) | **0.008** |  |
| ***MRI*** |  |  |  |  |  |
| Length of Spinal lesions^d^ |  |  |  |  |  |
| Detailed data available, n (%) ^e^ | 199 (80.1) | 104 (77.6) | 95 (84.1) | 0.259 |  |
| First attack, median (IQR) | 5.0 (3.0-8.0) | 5.0 (3.0-6.375) | 6.0 (4.0-9.0) | **0.004** |  |
| At last follow up, median (IQR) | 6.0 (4.0-9.0) | 6.0 (4.0-8.0) | 6.0 (4.0-11.0) | 0.061 |  |
| Brain lesions, n (%) |  |  |  |  |  |
| NMOSD typical lesion | 103 (41.7) | 63 (46.3) | 40 (35.4) | **0.071** |  |
| Area postrema | 26 (10.5) | 21 (15.7) | 5 (4.4) | **0.006** |  |
| Brainstem/cerebellum | 56 (22.7) | 37 (27.6) | 19 (16.8) | **0.048** |  |
| Adjacent to 3^rd^ ventricle | 30 (12.1) | 20 (14.9) | 10 (8.8) | 0.173 |  |
| Surrounding lateral ventricles | 32 (13.0) | 16 (11.9) | 16 (14.2) | 0.704 |  |
| Pyramidal tracts involvement | 17 (6.9) | 10 (7.5) | 7 (6.2) | 0.803 |  |
| Extensive hemispheric lesions | 8 (3.2) | 5 (3.7) | 3 (2.7) | 0.730 |  |

NMOSD: neuromyelitis optica spectrum disorder;EO: early-onset; LO-NMOSD: late-onset; SD: stand deviation; IQR: inter-quartile range; ON: optic neuritis; TM:transverse myelitis; ARR: annualized relapse rate; ARR-1: ARR excluding the first attack; EDSS: Expanded Disability Status Scale; PE: plasma exchange; IVIG: intravenous immunogloblin; IST: immunosuppressive therapy; AQP4-IgG: aquaporin-4 immunoglobin G; CSF: cerebrospinal fluid; OCB: oligoclonal band.

^a^ Combined syndrome: defined as a combination of two or more core clinical characteristics.

^b^ Proportions of symptoms involved during the entire disease duration.

^c^ Visual acuity For the visual comparison, only those patients who had at least 1 optic neuritis attack were considered.

^d^ Length of the spinal cord lesions were measured in terms of the number of vertebral segments.

^e^ For the comparison of length of spinal cord lesions, only those patients who had at least 1 transverse myelitis attack were considered.

Table 2 Comparison of demographic and clinical characteristics between RLO-NMOSD and VLO-NMOSD in patients with AQP4-IgG

|  | **RLO-NMOSD**  **(n=100)** | **VLO-NMOSD**  **(n=13)** | ***p* Value** |
| --- | --- | --- | --- |
| **Demographic** |  |  |  |
| Female, n (%) | 91 (91.0) | 10 (76.9) | 0.142 |
| Age onset, year, Mean ± SD | 57.86 ± 6.1 | 74.5 ± 3.6 | **0.000** |
| Disease duration, month, Mean ± SD | 35.8 ± 32.0 | 9.9 ± 8.8 | **0.000** |
| **Symtoms** |  |  |  |
| Symtoms at onset, n (%) |  |  |  |
| ON | 26 (26.0) | 1 (7.7) | 0.185 |
| TM | 56 (56.0) | 12 (92.3) | **0.014** |
| Brainstem+Cerebral | 9 (9.0) | 0 (0.0) | 0.595 |
| Combined ^a^ | 10 (10.0) | 1 (7.7) | >0.999 |
| Symptoms involvement, n (%) ^b^ |  |  |  |
| ON | 38 (38.0) | 3 (23.1) | 0.369 |
| TM | 88 (88.0) | 12 (92.3) | >0.999 |
| Brainstem+Cerebral | 36 (36.0) | 3 (23.1) | 0.537 |
| ON+TM ^a^ | 34 (34.0) | 3 (23.1) | 0.541 |
| **Relapses** |  |  |  |
| Recurrent, n (%) | 74 (74.0) | 8 (61.5) | 0.340 |
| Total number of relapses, Mean ± SD | 2.6 ± 1.4 | 1.9 ± 0.9 | 0.116 |
| Time to first relapse, month, Mean ± SD | 11.0 ± 10.6 | 9.5 ± 4.7 | 0.698 |
| ARR, Mean ± SD | 1.3 ± 0.9 | 1.3 ± 0.8 | 0.891 |
| ARR-1, Mean ± SD | 0.6 ± 0.5 | 0.6 ± 0.5 | 0.948 |
| **Disability** |  |  |  |
| EDSS at last follow up, median (IQR) | 3.5 (2.0-6.5) | 4.0 (3.0-7.5) | 0.128 |
| EDSS＞6 at last follow up, n (%) | 28 (28.0) | 6 (46.2) | 0.206 |
| EDSS＞8 at last follow up, n (%) | 11 (11.0) | 3 (23.1) | 0.203 |
| Visual acuity<0.1, n (%) ^c^ | 17 (17.0) | 2 (15.4) | >0.999 |
| Death, n (%) | 2 (2.0) | 1 (7.7) | 0.390 |
| **Treatment** |  |  |  |
| Acute phase treatment, n (%) |  |  |  |
| Glucocorticoid | 100 (100.0) | 11 (86.4) | **0.012** |
| PE or IVIG | 42 (42.0) | 8 (61.5) | 0.239 |
| Chronic treatment of IST, n (%) | 77 (77.0) | 6 (46.2) | **0.039** |
| Mycophenolate Mofetil | 64 (64.0) | 6 (46.2) | 0.236 |
| Azathioprine | 8 (8.0) | 0 (0.0) | 0.593 |
| Tacrolimus | 3 (3.0) | 0 (0.0) | >0.999 |
| Rituximab | 3 (3.0) | 0 (0.0) | >0.999 |
| **Lab** |  |  |  |
| Autoimmune antibodies, n (%) | 40 (40.0) | 7 (53.8) | 0.381 |
| Autoimmune diseases comorbidity, n (%) | 21 (21.0) | 2 (15.4) | >0.999 |
| Presence of OCB in CSF, n (%) | 4 (6.6) | 1 (9.1) | 0.581 |
| OCB unavailable, n (%) | 40 (40.0) | 2 (15.4) | 0.127 |
| Pleocytosis, n (%) | 23 (23.0) | 3 (23.1) | >0.999 |
| Increased CSF protein level, n (%) | 26 (26.0) | 7 (53.8) | **0.052** |
| **MRI** |  |  |  |
| Length of Spinal lesions^d^ |  |  |  |
| Detailed data available, n (%) ^e^ | 83 (83.0) | 12 (92.3) | 0.689 |
| First attack, median (IQR) | 6.0 (3.0-9.0) | 8.5 (6.5-13.0) | **0.018** |
| At last follow up, median (IQR) | 6.0 (4.0-9.75) | 11.0 (6.5-14.0) | **0.009** |
| Brain lesions, n (%) |  |  |  |
| NMOSD typical lesion | 37 (37.0) | 3 (23.1) | 0.376 |
| Area postrema | 5 (5.0) | 0 (0.0) | >0.999 |
| Brainstem/cerebellum | 19 (19.0) | 1 (7.7) | 0.458 |
| Adjacent to 3^rd^ ventricle | 10 (10.0) | 1 (7.7) | >0.999 |
| Surrounding lateral ventricles | 13 (13.0) | 3 (23.1) | 0.392 |
| Pyramidal tracts involvement | 7 (7.0) | 0 (0.0) | >0.999 |
| Extensive hemispheric lesions | 3 (3.0) | 0 (0.0) | >0.999 |

NMOSD: neuromyelitis optica spectrum disorder; RLO: very late onset; VLO: relative late onset; SD: stand deviation; IQR: inter-quartile range; ON: optic neuritis; TM:transverse myelitis; ARR: annualized relapse rate; ARR-1: ARR excluding the first attack; EDSS: Expanded Disability Status Scale; PE: plasma exchange; IVIG: intravenous immunogloblin; IST: immunosuppressive therapy; AQP4-IgG: aquaporin-4 immunoglobin G; CSF: cerebrospinal fluid; OCB: oligoclonal band.

^a^ Combined: defined as a combination of two or more core clinical characteristics.

^b^ Proportions of symptoms involved during the entire disease duration.

^c^ Visual acuity For the visual comparison, only those patients who had at least 1 optic neuritis attack were considered.

^d^ Length of the spinal cord lesions were measured in terms of the number of vertebral segments.

^e^ For the comparison of length of spinal cord lesions, only those patients who had at least 1 transverse myelitis attack were considered.
